# Evidence of a Genetic Basis for Differences in Parasitization Success between Strains of *Avetianella longoi* (Siscaro)

**DOI:** 10.1371/journal.pone.0129558

**Published:** 2015-06-08

**Authors:** Colin Umeda, Timothy D. Paine

**Affiliations:** Department of Entomology, University of California Riverside, Riverside, California, United States of America; University of Innsbruck, AUSTRIA

## Abstract

When the cerambycid, *Phoracantha recurva*, invaded California in the mid 1990’s a parasitoid wasp was imported from its native range in Australia as part of a biological control program. The wasp was later identified to be *Avetianella longoi*, which had already been released years earlier to control the congener longhorned beetle, *Phoracantha semipunctata*. Despite being recognized as the same species, the two wasps exhibited differential success on *P*. *recurva* eggs, indicating the presence of two separate strains. Here we determine if the differentiating factor between the two strains of *A*. *longoi* is a heritable genetic trait. All four pairings between the two strains were conducted, resulting in two homogenous and two heterogeneous crosses. All crosses except one produced viable F1 female offspring. F1 females were allowed to oviposit on *P*. *recurva* eggs and the survival of their offspring was compared to determine if survival can be affected by paternal contributions. The result was that the offspring of females with fathers from the second introduced strain showed significantly increased survival compared to F1 females with parents from the first introduced strain. This increased survival demonstrated that there is a heritable dominant trait that is associated with increased survival on *P*. *recurva* host eggs.

## Introduction

Hymenopteran endoparasitoids spend at least a portion of their development within the body of their host and utilize this host as their source of nourishment. For this strategy to be successful, the parasitoid must avoid being killed by the host’s innate immune response. The host’s immune response can be bypassed in multiple ways. Eggs can evade being detected due to coatings acquired from the mother [1, 2]. Immune responses can be suppressed by injected material, such as venom or polydnaviruses (PDVs), which causes the alteration of host characteristics or blocks immune pathways [3, 4, 5, 6].

These mechanisms of circumventing host immune responses can be passed on to the next generation in either through parental DNA or through vertical transmission of associated organisms. Parental genes control the formation of proteins that comprise venom. Polydnaviruses associated with suppression of immune responses are not actually viruses but are rather components of the wasp genome [7]. Vertical transmission of symbionts does not involve a genetic contribution from the parents. Defenses such as ascoviruses are mechanically transmitted by the wasp during oviposition as the female moves from parasitizing infected to uninfected hosts [8, 9, 10]. An exception to this would be the *Diadromus puchellus* ascovirus 4a which is in a mutualistic relationship with its wasp host and is transferred through vertical transmission from the mother and by ingestion of the virus by the developing wasp larvae [8, 10]. Cytoplasmic elements, such as bacterial endosymbionts, are also passed from the mother to the offspring and are not affected by paternal contributions; however, there is no evidence that cytoplasmically transmitted elements assist in suppressing the host immune response.

*Avetianella longoi* Siscaro (Hymenoptera: Encyrtidae) is an egg parasitoid of the Eucalyptus longhorned borers, *Phoracantha semipunctata* F. and *Phoracantha recurva* Newman (Coleoptera: Cerambycidae). It was originally introduced into California to control *P*. *semipunctata*, which was killing large numbers of *Eucalyptus* trees in California [11,12]. The introduction of the wasp for biological control was a complete success and reduced tree mortality caused by the beetle to negligible levels [13]. However, *P*. *semipunctata* was later replaced by its congener *P*. *recurva* which was a much less preferred host for *A*. *longoi* [14,15].

Unequal parasitization among the two beetle hosts is likely due to the encapsulation response that occurs in eggs of *P*. *recurva* but not in the eggs of *P*. *semipunctata* [16]. This immune response made the first strain of introduced *A*. *longoi* (S strain) a less than ideal agent to control the populations of *P*. *recurva*. Consequently, a second strain of *A*. *longoi* (R strain) was brought to California to control *P*. *recurva* [17]. This second strain exhibits significantly higher levels of survival on *P*. *recurva* compared to the first strain (C.U., unpublished data).

It is uncertain what physiological change occurred in the *A*. *longoi* R strain that allowed it to utilize *P*. *recurva* more efficiently than the S strain. Both hosts for *A*. *longoi* occur sympatrically throughout their native range in Australia [18] and the areas that each strain of wasps was collected were also located relatively close together [19, 17] so gene flow is a potential occurrence. The change is not due to phenotypic plasticity as the S strain was unable to change its performance even after being reared on *P*. *recurva* for 15 generations [15]. No information exists on the mechanism that facilitates higher survival of *A*. *longoi* on *P*. *recurva*, nor for how this trait is transmitted, whether genetically or otherwise, to the following generation.

Before tests of heritability can be made, it must be verified that the two strains can produce viable hybrids. Since *A*. *longoi* is haplodiploid it is relatively simple to determine if a successful mating has taken place. Haplodiploidy is a system where females are produced from fertilized eggs, whereas males are produced from unfertilized eggs. If the hybrid crosses yield females then the two strains can successfully interbreed. The objective of this study was to determine if the two strains of *A*. *longoi* successfully hybridize. Secondly, we examined if hybrid offspring exhibited increased survival on *P*. *recurva* compared to the S strain. Should hybrids have greater survival on *P*. *recurva*, it would suggest that the ability for the R strain to *A*. *longoi* is overcome the host immune response is heritable.

## Materials and Methods

### Insect rearing procedures

Colonies of *P*. *recurva* were maintained based on established procedures [11,12]. Adult beetles were kept in cages comprised of a cylinder of wire mesh with the top and bottom covered by 14.5 cm Petri dishes. The bottom of each cage was covered with a piece of wax paper. A 9 cm Petri dish covered with wax paper was placed in the bottom of the cage as a food container. Beetles were fed *ad libitum* with *Eucalyptus* pollen (Jarra Tree Pollen, Great Health Co., Brea, CA) and provided with a vial of 10% sucrose solution plugged with a dental wick. Each cage contained five females and three males to allow for multiple mating without increasing the chance of fights between males. The colony was maintained at approximately 27°C and 35% R.H. The photoperiod was 16 L: 8 D under full spectrum artificial lighting conditions.

Cage bottoms, feeding dishes, and associated wax paper were replaced every other day. Eggs were harvested from the wax paper or from between the two Petri dishes. These eggs were used to maintain the beetle colony and provide hosts for the parasitoid wasp colony.

Colonies of both strains of *A*. *longoi* were reared based on the procedures of [12] and each colony was housed in separate buildings to avoid cross-contamination. The two strains are morphologically and genetically indistinguishable so that it would be impossible to detect contamination if it were to occur. The S strain colony was kept in an insectary room in the laboratory, but the R strain was kept in the UCR Insectary and Quarantine facility.

Wasps were kept in cylindrical plastic cages that contained a mixture of adults of different ages and sexes. The top of each cage had a small hole that was covered by a fine mesh screen that had honey streaked across it to supply the wasps with food. Each cage was supplied with multiple egg cards consisting of egg masses of *P*. *recurva* that were oviposited no longer than two days prior. Egg masses were sandwiched between two pieces of wax paper to simulate natural oviposition beneath exfoliating *Eucalyptus* bark. The eggs were replaced approximately every 48 hours and the removed egg cards were placed into dated Petri dishes that were kept at ambient laboratory temperature. After adult emergence occurred, generally around 16 days post-oviposition, adult wasps were transferred over to the cylindrical cages.

### Hybrid Cross tests

To determine whether the two strains of *A*. *longoi* are able to successfully cross and produce fertile hybrid females, reciprocal crosses involving virgin males and females of each strain were conducted. *P*. *recurva* eggs containing wasp pupae were collected and placed individually into glass culture tubes, supplied with a small amount of honey and water solution (approximately 1:1), and plugged with cotton before emergence to ensure that all wasps used in this experiment were virgins.

One pair of wasps was placed in each glass culture tube supplied with a streak of 1:1 honey-water solution within 48 hours of their emergence. The wasp pairings were S♀S♂, S♀R♂, R♀S♂, and R♀R♂. Wasps were allowed to mate for 24 hours. After the mating period, the wasps were given access to 20 *P*. *recurva* eggs on a wax paper card for 48 hours for oviposition. The egg cards were then transferred into separate culture tubes and allowed to develop under ambient conditions. The numbers of males and females emerging from each egg card were counted and the presence or absence of female offspring was recorded. Each cross was replicated a minimum of 62 times.

To verify mating and sperm transfer, a single pair of each cross type was placed in an 11 mm diameter arena and observed for 40 minutes to determine if mating occurred. After 40 minutes, the wasp pair was transferred to a vial and held for an 24 hours, followed by a 48 hour oviposition period as described above. After the ovipoistion period, females were crushed under a glass microscope slide and the spermatheca was examined under a light microscope to determine if sperm was present. This process was replicated 18 times per cross.

To establish that mating incompatibility was not due to the presence of *Wolbachia*, both strains of *A*. *longoi* were screened using eubacterial primers fD1/rP2 from Weisberg et al. [20] and more *Wolbachia* specific primers 76F/1013R from O’Neill et al. [21] using *Wolbachia* sample PR09-605 obtained from the Stouthamer laboratory as the positive control.

### Hybrid Survival Test

To determine if increased survival on *P*. *recurva* eggs is a heritable trait, offspring survival for hybrid *A*. *longoi* females was compared to that of the pure *A*. *longoi* strains. Virgin females were obtained by isolating pupae from colony egg cards prior to emergence and virgin hybrid females were obtained by isolating pupae from egg cards from the appropriate cross. Within 48 hours of emergence, females were given ten fresh *P*. *recurva* eggs for oviposition. Wasps were allowed to oviposit for 24 hours before removing the egg card and replacing it with another ten fresh *P*. *recurva* eggs. For each egg card, the number of pedicels protruding from each egg were counted and then allowed to develop at ambient temperature and light. Eggs were monitored daily for the presence of black wasp pupae, which indicated emergence within the next few days. Following the appearance of black pupae, the number of adult male wasps that had emerged from parasitized eggs was checked twice a day. There were 45 S♀S♂, 33 S♀R♂, and 46 R♀R♂ strain virgin females tested and each strain was given a total of 900, 660, and 920 eggs, respectively.

Differences between the mean survival among the three treatments were determined by ANOVA with Least Squares Means separation at α≤0.05 [22].

## Results and Discussion

### Hybrid Cross Test

Copulation behaviors were observed while monitoring representative pairs in all four crosses. There were no observable differences in male or female behaviors regardless of the cross. Examination of spermathecae verified the presence of sperm in females from all four crosses. In all same strain crosses and crosses between S strain females and R strain males female offspring were consistently generated. Crosses between R strain females and S strain males did not produce female offspring.

### Hybrid Survival Test

**Fig 1:**
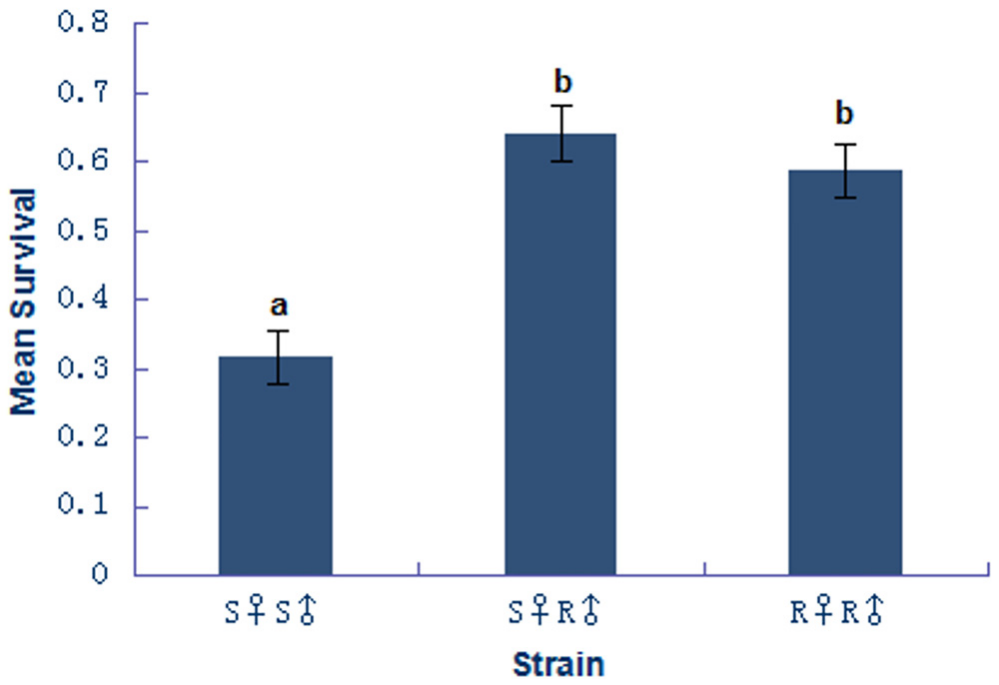
Proportion survival of *A*. *longoi (mean and s*.*e*.*)*. Strain lists female parent followed by male. Bars with different lowercase letters indicate that means were significantly different.

Survival trials did not include the R♀S♂ since this cross did not produce female offspring. Of the eggs provided to each strain the S♀S♂ strain parasitized 301/900 (33.44%) of the eggs, the S♀R♂ strain parasitized 510/660 (77.27%) of the eggs, and the R♀R♂ strain parasitized 710/920 (77.17%) of the eggs. There were significant differences in the mean survival of *A*. *longoi* with respect to strain (F_2,153_ = 3.02, p = 0.002) (Fig 1). The average survival for S♀S♂ strain offspring was 31.65%. For the hybrid S♀R♂ strain and the RR strain their mean survival was 64.12% and 58.64%, respectively. The SS strain exhibited significantly lower survival compared to both the S♀R♂ strain (p = 0.0002) and the RR strain (p = 0.004). The S♀R♂ strain exhibited significantly higher survival than the SS strain, but was not significantly different from the RR strain (p = 0.41).

Incompatibility between strains of insects can be due to multiple different barriers. There do not appear to be pre-zygotic barriers between *A*. *longoi* strains causing incompatibility. The geographic ranges of the cerambycid hosts overlap and the wasps were collected at sites not separated by any significant distances or barriers suggesting that there are not any temporal or spatial barriers. Ecological differences can promote sympatric speciation [23]; however, the strains of *A*. *longoi* do not exhibit differences in host preference [15]. The females appeared to accept males at similar rates and did not exhibit any difference in behavior towards male attempts to copulate which makes it unlikely that there are any behavioral barriers. Finally the males are able to copulate and transfer sperm to the spermatheca successfully which eliminates mechanical barriers. Consequently, it is likely that the observed asymmetric incompatibility is post-zygotic in nature.

Post-zygotic barriers can be either intrinsic or extrinsic. Extrinsic barriers are due to the hybrids being of an intermediate phenotype that is less fit than either parent. Intrinsic barriers would appear in the form of hybrid sterility or hybrid inviability. Since the R♀S♂ hybrids do not generate any female offspring, intrinsic hybrid inviability is the likely post-zygotic barrier.

Hybrid inviability is generated through a mismatch of genetic or cytoplasmic factors. Dobzhansky [24] and Muller [25] argued the interaction between genes or incompatibility between genes in hybrids in different population is what causes isolation. Haldane’s rule [26] predicts that the inviable sex in hybrids resulting from incompatible crosses will be the heterogametic sex. Haldane’s rule could still be applied to systems that are haplodiploid if the female sex is considered to be heterogametic due to the fact that they are receiving alleles from both parents (even if they are both X chromosomes), especially since males are hemizygous and only receive contributions from one parent which would leave them unable to form combinations of genes that could be incompatible.

The mismatch of genes could be nuclear-nuclear, nuclear-cytoplasmic, or nuclear-mitochondrial. Nuclear-nuclear effects are derived from the genetic contributions of the mother and father not being compatible, whether through modifications in pathways, sequence divergence leading to problems with recombination, or key genes being moved or absent on the chromosomes [27]. With nuclear-nuclear incompatibility it would be expected that incompatibility will occur in both directions. Since cytoplasmic and mitochondrial elements are passed down directly from the mother to the offspring incompatibility between the parents is not necessarily going to occur in both directions. It is possible to observe unidirectional incompatibility when only one strain has a cytoplasmic or mitochondrial type that is incompatible with another type. It has been shown that there can be a conflict between nuclear and cytoplasmic elements which results in incompatibility [28]. We use the word “elements” and “type” due to the existence of bacteria, such as *Wolbachia*, that commonly cause incompatibility. *Wolbachia* is relatively widespread and infection causes unidirectional incompatibility where infected males are unable to create hybrids with uninfected females or females with different types of *Wolbachia* infections [29]. However, in this study, both the eubacterial and *Wolbachia* specific tests were negative for the presence of a bacterial endosymbiont that might be affecting the strains ability to successfully breed in both directions.

The results of the hybrid crosses demonstrated that the two strains of *A*. *longoi* will interbreed, but suffer from unidirectional incompatibility. Hybrid crosses between R strain females and S strain males only produce males, which are the hemizygous sex and do not possess a combination of genetic material. As Haldane’s rule predicts only the heterogametic sex is negatively affected in the incompatible cross. Furthermore, the incompatibility only occurs in one direction which makes it unlikely that the mismatch is due to nuclear-nuclear interactions. Due to this, the cause for the unidirectional incompatibility could be the result of negative effects stemming from cytoplasmic elements or conflicts between the mitochondrial genes and the nuclear genes.

Should there be some sort of cytoplasmic or mitochondrial incompatibility, it is likely that the element in question is resident within the S strain. This conclusion is based on the observation that the introduction of genetic material from the R strain yields no females when their maternally inherited material is combined with the genetic material of the S strain, but the genetic material of the R strain is able to mix with the maternally inherited material of the S strain and produce viable fertilized eggs. It is uncertain whether females will exhibit a preference for one strain over the other as both strains of females mated with both strains of males. A preference for same strain males would enhance individual fitness, at least for the R strain, because it allows the female to avoid wasting time and resources on matings that will produce no female offspring.

## Conclusions

Based on the higher rate of survival in the hybrid strain compared to the S strain it indicates that avoiding encapsulation in *P*. *recurva* is due to a heritable difference in the R strain that the S strain lacks. If the difference was not heritable then we would expect to see hybrids that perform similar to their maternal strain. What is witnessed, however, is survival in hybrids that was similar to the paternal R strain of *A*. *longoi* and significantly greater than the maternal S strain. It is possible to rule out the differentiating factor between the two strains, with respect to the immune response of *P*. *recurva*, being due to the presence of an immune triggering factor in the S strain as the S♀R♂ strain would inherit this factor and provoke an immune response like their mother. Also, if the difference between the strains is due to cytoplasmic factors, then the S♀R♂ strain would have a survival rate similar to the S strain as cytoplasmic elements are transmitted from mother to offspring and not dependent on any paternal contribution.

This asymmetry implies that cytoplasmic elements do not play a role in overcoming host immunity as the R strain males do not contribute any cytoplasmic elements to their offspring and yet the S♀R♂ strain still had significantly higher survival than its maternal S strain. Since survival was increased after a paternal contribution from the R strain it is likely that the R strain contains a different gene or an alternate form of an allele that separates the two strains with respect to utilizing *P*. *recurva*. Due to the increase in survival in the F1 generation it would appear that if the difference between strains is an allele, then it is not recessive as it was able to affect performance in a heterozygous form. It is still uncertain what has changed within the R strain to allow them to develop and survive better in the eggs of *P*. *recurva*.
